# Winding around non-Hermitian singularities

**DOI:** 10.1038/s41467-018-07105-0

**Published:** 2018-11-15

**Authors:** Qi Zhong, Mercedeh Khajavikhan, Demetrios N. Christodoulides, Ramy El-Ganainy

**Affiliations:** 1 Department of Physics and Henes Center for Quantum Phenomena, Michigan Technological University, Houghton, MI 49931 USA; 2 College of Optics & Photonics-CREOL, University of Central Florida, Orlando, FL 32816 USA; 3 Max Planck Institute for the Physics of Complex Systems, Nöthnitzer Strasse 38, 01187 Dresden, Germany

## Abstract

Non-Hermitian singularities are ubiquitous in non-conservative open systems. Owing to their peculiar topology, they can remotely induce observable effects when encircled by closed trajectories in the parameter space. To date, a general formalism for describing this process beyond simple cases is still lacking. Here we develop a general approach for treating this problem by utilizing the power of permutation operators and representation theory. This in turn allows us to reveal a surprising result that has so far escaped attention: loops that enclose the same singularities in the parameter space starting from the same point and traveling in the same direction, do not necessarily share the same end outcome. Interestingly, we find that this equivalence can be formally established only by invoking the topological notion of homotopy. Our findings are general with far reaching implications in various fields ranging from photonics and atomic physics to microwaves and acoustics.

## Abstract (web summary)

A general description of observable effects induced by non-Hermitian singularities is complex. Here, Zhong et al. develop such a formalism, showing that loops around the same exceptional point starting from the same point in the same direction do not need to have the same outcome.

## Introduction

Non-Hermitian singularities arise in multivalued complex functions^1,2^ as points where the Taylor series expansion fails. In the context of non-Hermitian Hamiltonians, these points, commonly referred to as exceptional points (EPs) feature special degeneracies where two or more eigenvalues along with their associated eigenfunctions become identical^3,4^. An EP of order *N* (EPN) is formed by *N* coalescing eigenstates. Recently, the exotic features of EPs have been subject of intense studies^5–8^ with various potential applications in laser science^9–12^, optical sensing^13–15^, photon transport engineering^16,17^, and nonlinear optics^18,19^ just to mention few examples. For recent reviews, see refs.^20,21^.

Very often, EPs are points of measure zero in the eigenspectra of non-Hermitian Hamiltonians, which makes them very difficult to access, even with careful engineering. Yet, their effect can be still felt globally. Particularly, an intriguing aspect of non-Hermitian systems is the eigenstate exchange along loops that trace closed trajectories around EPs. In this regard, stroboscopic encircling of EP2 (EP of order 2) has been studied theoretically^22,23^ and demonstrated experimentally in various platforms such as microwave resonators^24,25^ and exciton-polariton setups^26^. Additionally, Berry’s phase around EPs has been also theoretically investigated in details^27–30^. Complementary to these efforts, the dynamic encircling of EPs was shown to violate the standard adiabatic approximation^31–34^. These predictions were recently confirmed experimentally by using microwave waveguides platforms^35^ and optomechanical systems^36^.

Notably, the aforementioned studies focused only on systems having only one EP of order two. Richer scenarios involving multiple and/or higher-order EPs have been largely neglected, with rare exceptions that treated special systems (admitting simple analytical solutions) on a case-by-case basis^37,38^. This gap in the literature is probably due to the complexity of the general problem and its perceived experimental irrelevance. However, recent progress in experimental activities that explore the physics of non-Hermitian systems are quickly changing the research landscape, and controlled experiments that probe more complicated structures with multiple EPs will be soon within reach. These developments beg for a general approach that can provide a deeper theoretical insight into these complex systems.

In this work, we bridge this gap by introducing a general formalism for treating the eigenstate exchange along arbitrary loops enclosing multiple EPs. More specifically, our approach utilizes the power of group theory together with group representations to decompose the final action of any loop into more elementary exchange processes across the relevant branch cuts (BCs). This formalism simplifies the analysis significantly, which in turn allows us to gain an insight into the problem at hand and unravel a number of intriguing results: trajectories that encircle the same EPs starting from the same initial point and having the same direction do not necessarily lead to an identical exchange between the eigenstates; establishing such equivalence between the loops (i.e. same eigenstate exchange) is guaranteed only by invoking the topological notion of homotopy. As a bonus, our approach can also paint a qualitative picture of the dynamical properties of the system.

## Results

### General formalism for encircling multiple EPs

**Fig. 1:**
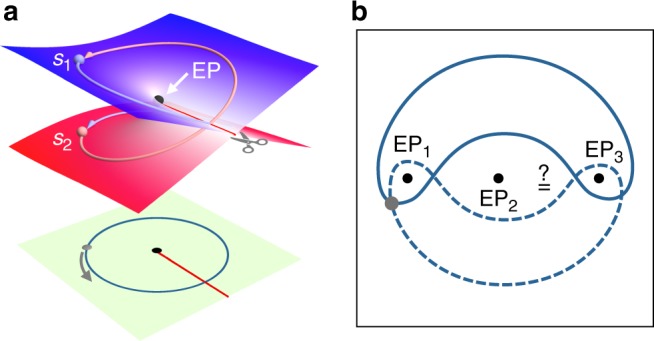
Different ways of encircling multiple expectational points (EPs). **a** Illustration of Riemann surface associated with the square root function associated with an archetypal 2 × 2 non-Hermitian Hamiltonian. A loop that encircles the EP (also known as the branch point) starting from the state *s*_1_ will map it onto *s*_2_ and vice versa. In the complex plane projection, this is represented by adding a branch cut as shown by the red line. The gray arrow in the projection plane indicates the encircling direction. **b** A scenario that exhibits three EPs. In this case, loops can encircle the same EPs in different ways as illustrated by the two loops (solid/dashed lines) that enclose EP_1,3_ starting from the same point (gray dot)

Before we start our analysis, we first describe the simple case of EP2. These are special points associated with the multivalued square root function in the complex plane. The Riemann surface of this function is shown in Fig. 1a. Clearly, as two parameters are varied in the complex plane to trace a closed loop, the initial point on the surface ends up on a different sheet. This process can be also viewed by considering the projection on the complex plane after adding a BC. As we mentioned before, this simple scenario has been studied in the literature in both the stroboscopic and dynamical cases. Consider however what happens in more complex situations where there are more than one EP. For instance, Fig. 1b depicts a case with three EPs. One can immediately see that this scenario exhibits an additional complexity that is absent from the previous case. Namely, there are now different ways for encircling the same EPs (as shown by the solid and dashed loops in the figure). This in turn raises the question as whether these loops lead to the same results or not. These are the type of questions that we would like to address in this work. As we will see, in resolving these questions, our analysis also reveals several peculiar scenarios.

**Fig. 2:**
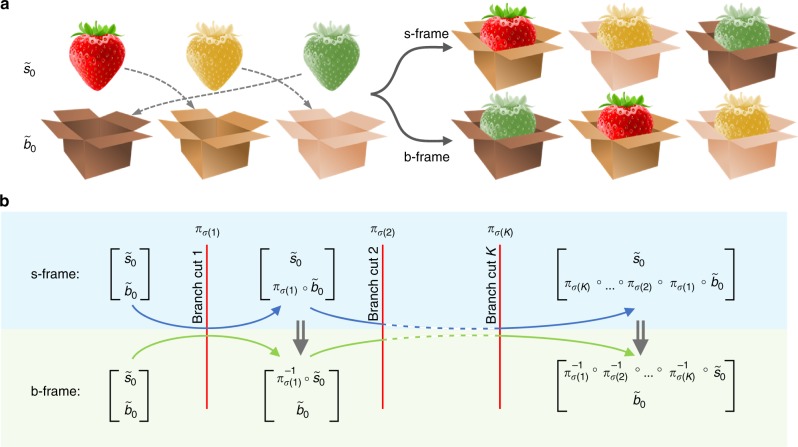
Different permutation frames. **a** A simple illustration of the two different frames used for representing the same configuration. **b** A summary of the mathematical formulation of the concept depicted in **a** (see main text for more details)

To this end, let consider an *n*-dimensional non-Hermitian discrete Hamilton. The Riemann surface associated with the real (or imaginary) part of its eigenvalues will consist of *n* sheets corresponding to different solution branches. We will label these *n* branches as *b*_1_, *b*_2_,…, *b*_*n*_. In the complex plane, these branches are separated by BCs. Thus, an initial point on any trajectory in the complex plane will correspond to *n* initial eigenstates, which we will label as *s*_1_, *s*_2_,…, *s*_*n*_. The eigenvalue for each state *s*_*i*_ will be denoted by *λ*_*i*_. As the encircling parameters are varied, the eigenstates will move along the trajectory, crossing from one branch to another across the BCs. The crucial point here is that we will always fix the initial subscript of the state as it changes. We now describe the initial configuration on the trajectory by the mapping:

1 $${\cal C}_0 = \left[ {\begin{array}{*{20}{c}} {\tilde s_0} \\ {\tilde b_0} \end{array}} \right],$$

where $\tilde s_0 = (s_1,s_2,...,s_n)$ and $\tilde b_0 = (b_1,b_2,...,b_n)$ are two ordered sets. In our notation, ${\cal C}_0$ maps (or associates) every element of $\tilde s_0$ to the corresponding element in $\tilde b_0$. Note that we can change the orders of the elements in both $\tilde s_0$ and $\tilde b_0$ identically without changing ${\cal C}_0$. In other words, we have several different ways for the same configuration. As the loop crosses BCs, the exchange between the eigenstates will result in new configurations, which, again, can be described in different ways. Two particular choices are interesting here. In the first one, we always fix $\tilde s_0$ and allow the elements of $\tilde b_0$ to shuffle, effectively creating a new $\tilde b$. In the second, we just do the converse. We will call these two equivalent notations the s- and b-frames, respectively. This is explained by the cartoon picture in Fig. 2a.

The first step in our analysis is to choose a scheme for sorting the eigenstates and locating the BCs accordingly. We will discuss the details of the sorting later but for now we assume that we have a certain number of BCs and we label each one with a unique integer value (positive for a crossing in certain direction and negative for reverse crossing). Next we determine how the eigenstates are redistributed across an infinitesimal trajectory across each BC (see discussion later on sorting schemes). For every loop, we then create an ordered list *σ* that contains the number of the crossed BCs in the order they are crossed by the loop. In other words, the element *σ*(*j*) is the number of the *j*-th crossed BC. Clearly, the set *σ* will be in general different from loop to another and even can be different for the same loop depending on the initial point or the encircling direction. Then the final configuration in both the s- and b-frames is given by:

2 $${\cal C}_\sigma ^{\mathrm{s}} = \left[ {\begin{array}{*{20}{c}} {\tilde s_0} \\ {\tilde b_\sigma } \end{array}} \right] \equiv \left[ {\begin{array}{*{20}{c}} {\tilde s_0} \\ {{\cal P}\left[ {{\prod} \pi _{\sigma (j)}} \right] \circ \tilde b_0} \end{array}} \right],$$

3 $${\cal C}_\sigma ^{\mathrm{b}} = \left[ {\begin{array}{*{20}{c}} {\tilde s_\sigma } \\ {\tilde b_0} \end{array}} \right] \equiv \left[ {\begin{array}{*{20}{c}} {\left\{ {{\cal P}\left[ {{\prod} \pi _{\sigma (j)}} \right]} \right\}^{ - 1} \circ \tilde s_0} \\ {\tilde b_0} \end{array}} \right],$$

where ${\cal P}$ denotes the ordering operator, which arranges the multiplication of the permutation operators *π*_*σ*(*j*)_ from right to left according to the order of crossing the BCs; and the product runs across the index *j*. For example, if *σ* = (3, 1, 2), then the ${\cal P}\left[ {{\prod} {\pi _{\sigma (j)}} } \right] = \pi _{\sigma (3)} \circ \pi _{\sigma (2)} \circ \pi _{\sigma (1)} = \pi _2 \circ \pi _1 \circ \pi _3$. The permutation operator *π*_*k*_ associated with BC *k* is the standard permutation mapping that, which when applied to a set, will shuffles the order of its elements^39^. Here it is used to describe how the eigenstates are redistributed when a trajectory crosses a BC. For instance, if the permutation exchange the order of the first two elements of $\tilde b_0$ across a BC *k*, then *π*_*k*_(*b*_1,2_) = *b*_2,1_, and *π*_*k*_(*b*_*i*_) = *b*_*i*_ for *i* > 2. Figure 2b illustrates the relation between the s- and b-frame calculations as expressed by Eqs. (2) and (3).

The above discussion can be directly mapped into linear algebra by using representation theory. To do so, we define the vectors **s**_0_ = (*s*_1_, *s*_2_,...,*s*_*n*_)^T^ and **b**_0_ = (*b*_1_, *b*_2_,...,*b*_*n*_)^T^. In the s-frame, we will fix **s**_0_ and allow **b** to vary in order to represent the change in configuration. In the b-frame, we just do the opposite. For instance, if after crossing a BC, eigenstate 1 moves to branch *n*, eigenstate 2 moves to branch 1, and eigenstate *n* moves to branch 2, this will be expressed as **b**_1_ = (*b*_*n*_, *b*_1_,...,*b*_2_)^T^ in the s-frame; and **s**_1_ = (*s*_2_, *s*_*n*_,...,*s*_1_)^T^ in the b-frame. After a loop completes its full cycle, the final vector is then compared with the initial one to determine the exchange relations between the eigenstates. For instance, if the above vector was the final result, the exchange relations will be: {*s*_1_, *s*_2_,..., *s*_*n*_} → {*s*_*n*_, *s*_1_,...,*s*_2_}, which means that after the evolution *s*_1_ became *s*_*n*_, *s*_2_ became *s*_1_, and *s*_*n*_ became *s*_2_.

We can now express the action of the permutation operators *π*_*k*_ by the matrices $P_{\pi _k}$ whose elements are obtained according to the rule $P_{\pi _k}(m,l) = 1$ if *b*_*l*_ = *π*_*k*_(*b*_*m*_), and 0 otherwise^40^. In the s- and b-frames, the redistribution of the eigenstates across the branches in Eqs. (2) and (3) can be then described by:

4 $${\mathbf{b}}_\sigma = \left\{ {{\cal P}\left[ {{\prod} M_{\sigma (j)}} \right]} \right\}^{ - 1}{\mathbf{b}}_0,$$

5 $${\mathbf{s}}_\sigma = {\cal P}\left[ {{\prod} M_{\sigma (j)}} \right]{\mathbf{s}}_0,$$

where $M_k = P_{\pi _k}^{ - 1}$. In arriving at the above equation, we have used standard results from group theory: $P_{\pi _2 \circ \pi _1} = P_{\pi _1}P_{\pi _2}$ and $P_{\pi ^{ - 1}} = P_\pi ^{ - 1}$.

In the rest of this manuscript, we adopted the b-frame with matrices *M*. This approach offers a clear advantage: the order of the matrices acting on the state vectors **s** is consistent with the order of crossing the BCs. As we will see shortly, this will allow us to develop the topological features of the equivalent loops in a straightforward manner. Finally, we note that if crossing a BC from one direction to another is associated with a matrix *M*, the reverse crossing will be described by *M*^−1^. In some cases (such as with EP2), we can have *M*^−1^ = *M* but this is not the general case.

Our discussion so far focused on developing the general formalism by assuming that the eigenstates of the system are somehow classified according to a certain criterion. This is equivalent to say that we divide the associated Riemann surface into different sheets, each harboring a solution branch. Of course, one can pick any such criterion to classify the solutions. In previous studies that involved one EP of order two or three, the eigenstates were classified based on the analytical solution of the associated characteristic polynomial. This however has two drawbacks: it generates relatively complex branches on the Riemann sheet; and it cannot be applied for discrete Hamiltonians having dimensions larger than four since analytical solutions do not exist for polynomials of order five or larger. Thus, our analysis above is useful only if one can find a sorting scheme that circumvents the above problems. Interestingly, such a sorting scheme is easy to find. Particularly, we can sort the eigenstates based on the ascending (or descending) order of the real or imaginary parts of their eigenvalues. This scheme can be easily applied to any system of arbitrarily high dimensions. Moreover, it lends itself to straightforward numerical implementations. To compute the permutation operator *π*_*k*_ and its associated matrix *M*_*k*_ across a BC *k*, one chooses an infinitesimal trajectory that crosses the BC and calculates how the eigenvalues evolve along this trajectory, comparing their order before and after crossing the BC. That will immediately provide information about the permutations. We illustrate this using a concrete example later.

### Equivalent loops and homotopy

Here we employ the predictive power of our formalism to address the following question: are there any global features that characterize the equivalence between different loops in the parameter space regardless of their geometric details? In answering this question, we will first focus on the stroboscopic case and later discuss the implication for the dynamical behavior.

Here two loops are called equivalent if they lead to identical static eigenstates exchange. It is generally believed that two similar loops in the parameter space starting at the same point and encircling the same EPs in the same direction are equivalent. Surprisingly, we will show below that this common belief is wrong.

In general two loops will be equivalent if they have the same matrix product in Eq. (5). This can occur for two unrelated loops which we will call accidental equivalence. However, we are particularly interested in establishing the conditions that guarantee this equivalence. To do so, we invoke the notion of homotopy between loops. In topology, two simple paths (a simple path is a one that does not intersect itself), having the same fixed end points in a space *S*, are called homotopic if they can be continuously deformed into each other^41^. If the two end points of a path are identical, this path is a loop with the identical end point as a basepoint. The space *S* here will be a two-dimensional (2D) punctured parameter space after removing all EPs. Based on these definitions, we can now state the main results of this section: homotopy is a sufficient condition for equivalence between loops; loops that are connected by free homotopy (continuous deformation between loops without any fixed points) can be equivalent for some starting points and inequivalent for others.

**Fig. 3:**
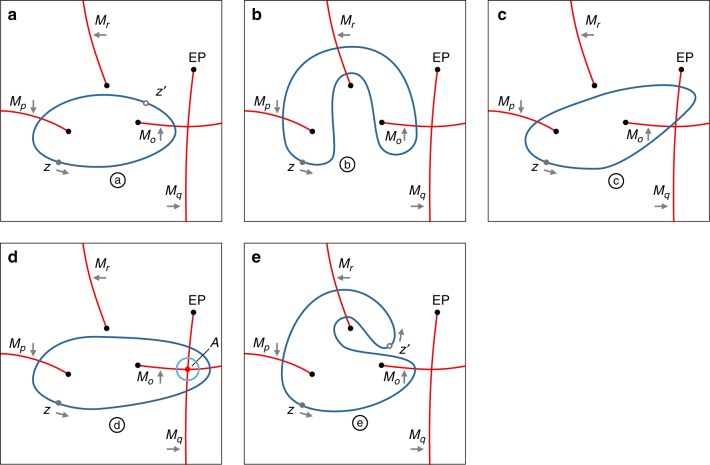
Homotopy between loops. Illustration of equivalence between homotopic loops in the parameter space of a generic Hamiltonian. **a** Loop a encloses two exceptional points (EPs) associated with matrices *M*_*o*_ and *M*_*p*_. **b** Loop ⓑ encloses the same two EPs yet it cannot be deformed into loop ⓐ without crossing EP associated with *M*_*r*_. Consequently, it has a different matrix product (assuming not accidental equivalence). On the other hand, loops ⓒ and ⓓ in **c** and **d** can be deformed into loop ⓐ without crossing any EP. As a result, they are equivalent (have the same matrix product) as shown in the text. **e** A peculiar case of free homotopy is presented. Loop ⓔ is homotopic with loop ⓐ for the starting point *z* but not for *z*′. As a result, the two loops are equivalent for the former point but not for the latter. The discussion here is very generic and can be extended easily to any other configuration of EPs and branch cuts (BCs). As a side note, we emphasize that the choice of the BCs is not unique. However, while different partitioning will lead to a new set of matrices, the final results and the topological relations between the loops are invariant. Black dots represent EPs, red lines are the BCs, and the blue loops are the encircling trajectories

In order to validate this statement, we consider a generic Hamiltonian having a number of EPs and, without any loss of generality, we focus only on a subset of the spectrum as shown in Fig. 3. The axes on the figures represent any two parameters of the Hamiltonian. We define the space *S* to be the 2D parameter space excluding the EPs. Figure 3a depicts a loop ⓐ that encircles two EPs starting from point *z* in the counterclockwise (CCW) direction. Consequently, its final permutation matrix is given by *M*_*p*_*M*_*o*_. Consider now what happens when loop ⓐ is deformed continuously to a new loop. Apart from the trivial case where the deformation does not change the number or the order of BC crossing, different interesting scenarios can arise as illustrated in Fig. 3b–e. Particularly, in Fig. 3b, the deformation can take place only by crossing additional EP, where it is clear that the new matrix product of loop ⓑ ($M_pM_rM_oM_r^{ - 1}$) is in general different than the initial one. In this case, the two loops are not necessarily equivalent (unless accidental equivalence takes place). In the case illustrated in Fig. 3c, the deformation can change the number of the crossed BCs in pairs traversed consecutively back and forth but without crossing any EP. Here the two loops ⓐ and ⓒ are also equivalent because the matrix product is still the same: $M_pM_q^{ - 1}M_qM_o = M_pM_o$. Alternatively, the deformation shown in Fig. 3d changes the number of the crossed BCs in pairs traversed back and forth but not consecutively. In this case, the final matrix product is given by $M_pM_q^{ - 1}M_oM_q$. It is not immediately clear if this product is equivalent to *M*_*p*_*M*_*o*_. However, since the intersection point of the BCs (point *A* in Fig. 3d) is not an EP, then by definition, encircling point *A* with a loop that does not enclose any EP must give the identity operator. In terms of matrices, this translates into $M_oM_qM_o^{ - 1}M_q^{ - 1} = I$, or [*M*_*o*_, *M*_*q*_] = 0, where *I* is the identity matrix. Consequently, $M_pM_q^{ - 1}M_oM_q = M_pM_q^{ - 1}M_qM_o = M_pM_o$, i.e. loops ⓓ and ⓐ are equivalent. Finally, we can also have a loop similar to ⓔ as shown in Fig. 3e. This probably the most intriguing situation. For a starting point at *z*, both loops ⓐ and ⓔ have the same matrix product *M*_*p*_*M*_*o*_, which is consistent with the fact that they can be deformed into one another without crossing any EP. On the other hand, for a different starting point such as *z*′, the matrix product of loop ⓔ is given by $M_r^{ - 1}M_oM_pM_r$, i.e. different than that of loop ⓐ, which is given by *M*_*o*_*M*_*p*_. Note that for this starting point, the two loops cannot be deformed into each other without crossing any EP. In topology, a continuous deformation that does not involve fixed points is called free homotopy. Here loops ⓐ and ⓔ are connected by free homotopy, i.e. they are in general equivalent only for a subset of all the possible starting points. This completes our argument.

The above discussion focused only on the stroboscopic case. However, as we will show in the explicit example presented in the next section, homotopy is also relevant to the dynamical encircling of EPs. Particularly, our numerical calculations show that homotopic loops can have the same outcome, despite the failure of the adiabatic perturbation theory. These results can be better understood by considering the evolution of the loops on the full three-dimensional (3D) Riemann surface as we further discuss later.

### Illustrative examples

We now discuss a concrete numerical example to demonstrate the application of our formalism and confirm the various predictions of the previous discussion. We emphasize that the example considered below is not a special case. It was merely chosen because it is complex enough to exhibit the exotic effects before, yet not too complex to impede physical implementations.

Consider the following Hamiltonian:

6 $$H = \left[ {\begin{array}{*{20}{c}} {i\gamma } & J & 0 & 0 \\ J & 0 & \kappa & 0 \\ 0 & \kappa & 0 & J \\ 0 & 0 & J & { - i\gamma } \end{array}} \right],$$

where *i* is the imaginary unit, *κ* and *J* are coupling coefficients, and *γ* is the non-Hermitian parameter. In what follows, the four eigenvalues of *H* will be investigated as a function of the complex *κ* by fixing *J* = *γ* = 1 (in certain physical platforms such as optics, it might be practically easier to fix all the parameters and change *γ*, but that will not affect the main conclusions of this work).

**Fig. 4:**
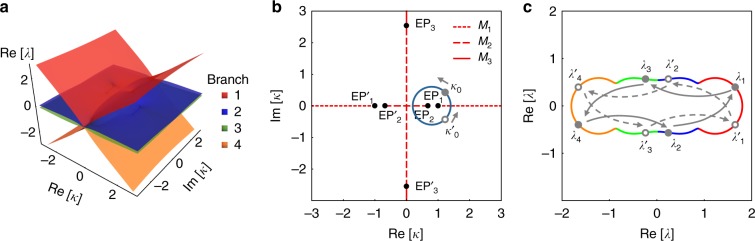
Numerical illustration of our approach. **a** The branches of Riemann surface of the real part of eigenvalues of *H* in Eq. (6) are distinguished by different colors according to the magnitude of Re[*λ*]. **b** The exceptional points (EPs) (black dots) and their corresponding branch cuts (BCs) (red lines) are illustrated. Each BC is related with a permutation matrix *M*_1,2,3_ in Eq. (7). One closed loop (blue line) encircles EP_1_ and EP_2_ counterclockwise (CCW), starting from *κ*_0_ or $\kappa _0^\prime$ (the solid or hollow gray points) on the loop. Loops intersecting with BCs would lead to eigenvalues moving from one branch to another, and result in the swap of eigenstates finally. **c** The stroboscopic evolution of complex eigenvalues are plotted as a parametric function of *κ* when it moves along the loop CCW. The eigenvalues at the starting point are labeled as gray points (solid or hollow) on their trajectory. The colors in the eigenvalue trajectory represent which branch the eigenvalues are located at instantaneously. The joints of two colors are where the *κ* crosses the BCs. The gray points (solid or hollow) and arrows illustrate the evolution of eigenvalues for starting from *κ*_0_ or *κ*′_0_, and therefore the evolution of eigenstates is {*s*_1_, *s*_2_, *s*_3_, *s*_4_} → {*s*_3_, *s*_1_, *s*_4_, *s*_2_} and →{*s*_2_, *s*_4_, *s*_1_, *s*_3_}, respectively

Under these conditions, *H* has three pairs of EPs at *κ* = ±1, $\pm \sqrt {2\sqrt 3 - 3}$, $\pm i\sqrt {2\sqrt 3 + 3}$, which we will denote by EP_1_, EP'_1_, EP_2_, EP'_2_, EP_3_, EP'_3_, respectively. In each group, EP'_1,2,3_ has same properties as EP_1,2,3_. The Riemann surface and the distribution of the EPs in the complex *κ* plane are shown in Fig. 4a, b, respectively.

As discussed previously, the first step in our approach is to identify a simple sorting method. Here we chose to sort the eigenvalues according to the magnitude of their real parts as shown in Fig. 4a, where every branch is distinguished by a distinct color. From this figure, we can also identify the features of the EPs as follows: EP_1_ and EP'_1_ are of second order and connect branches 2 and 3; EP_2_ and EP'_2_ are of second order and connect branches 1 and 2 on one hand, and branches 3 and 4 on the other; and finally EP_3_ and EP'_3_ are of second order and connect branches 1 and 3 as well as branches 2 and 4 (in fact all the four surfaces of Re[*λ*] are connected at EP_3_ and EP'_3_, and one has to look at the Im[*λ*] surface to infer the connectivity). Equivalently, the surface connectivity across the EPs can be characterized by using a 2D plane spanned by the real and imaginary parts of *κ* along with the BC lines that separate the different solution branches and the information on the transition between the different branches across each line. The latter can be expressed in terms of permutation matrices. Our sorting scheme of the eigenvalues of *H* results in six BCs as shown in Fig. 4b, but one can identify only three different permutation matrices:

7 $${\begin{array}{*{20}{l}} {M_1} \hfill & { = \left[ {\begin{array}{*{20}{c}} 1 & 0 & 0 & 0 \\ 0 & 0 & 1 & 0 \\ 0 & 1 & 0 & 0 \\ 0 & 0 & 0 & 1 \end{array}} \right], M_2 = \left[ {\begin{array}{*{20}{c}} 0 & 1 & 0 & 0 \\ 1 & 0 & 0 & 0 \\ 0 & 0 & 0 & 1 \\ 0 & 0 & 1 & 0 \end{array}} \right], M_3 = \left[ {\begin{array}{*{20}{c}} 0 & 0 & 0 & 1 \\ 0 & 0 & 1 & 0 \\ 0 & 1 & 0 & 0 \\ 1 & 0 & 0 & 0 \end{array}} \right].} \hfill \end{array}}$$

The correspondence between these matrices and the BCs is depicted in Fig. 4b. It is not difficult to see that the above matrices have the following properties: $M_1^2 = M_2^2 = M_3^2 = I$, and [*M*_1_, *M*_3_] = [*M*_2_, *M*_3_] = 0.

We now focus on the stroboscopic encircling of EPs. As illustrative example, we consider the loop encircling both EP_1_ and EP_2_, as shown in Fig. 4b. Clearly, the final exchange relation is determined by the product of *M*_1_ and *M*_2_. Since [*M*_1_, *M*_2_] ≠ 0, one has to be more specific about the starting point and direction. For sake of illustration, let us choose CCW direction, and *κ*_0_ or *κ*′_0_ as the starting point. In the first case, the loop intersects the BC associated with *M*_2_ first before it crosses that of *M*_1_. As such, we have *M*_1_*M*_2_(*s*_1_, *s*_2_, *s*_3_, *s*_4_)^T^ = (*s*_2_, *s*_4_, *s*_1_, *s*_3_)^T^, which in turn implies the exchange {*s*_1_, *s*_2_, *s*_3_, *s*_4_} → {*s*_3_, *s*_1_, *s*_4_, *s*_2_}. Similarly, the starting point *κ*′_0_ will give *M*_2_*M*_1_(*s*_1_, *s*_2_, *s*_3_, *s*_4_)^T^ = (*s*_3_, *s*_1_, *s*_4_, *s*_2_)^T^, which leads to {*s*_1_, *s*_2_, *s*_3_, *s*_4_} → {*s*_2_, *s*_4_, *s*_1_, *s*_3_}. These exchange relations are also evident from the eigenvalue trajectories in Fig. 4c. Another important consequence for the absence of commutation between *M*_1_ and *M*_2_ is that *M*_2_*M*_1_*M*_2_*M*_1_ ≠ *I*. Hence encircling the loop in Fig. 4b twice still lead to nontrivial exchange. For example, the state *s*_1_ will evolve into *s*_3_, *s*_4_, and *s*_2_ after encircling the loop one, two, and three times, respectively. We have also confirmed (not shown here) that our formalism can produce the results for the 3 × 3 Hamiltonians, where encircling two EPs of order two can equivalent to encircling a third-order EP^37,38,42^.

**Fig. 5:**
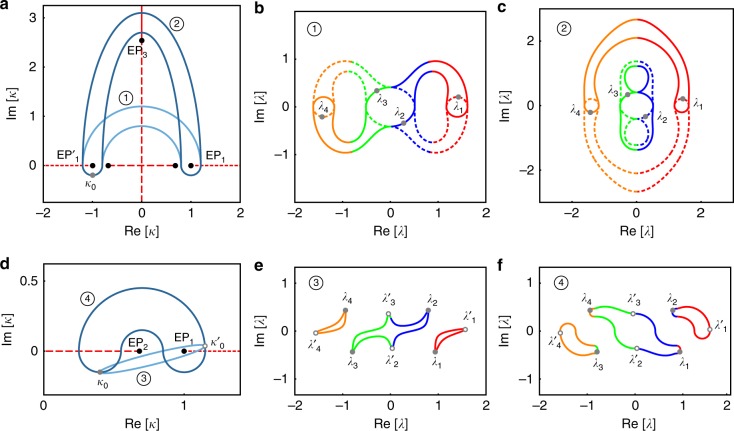
Numerical example of homotopic relations between loops. **a** Two similar loops ${\bigcirc{{\hskip -6.7pt}{\scriptstyle{1}}}}{\hskip 2pt}$ and ${\bigcirc{{\hskip -6.7pt}{\scriptstyle{2}}}}{\hskip 2pt}$ encircle the exceptional points EP_1_ and EP'_1_. The two loops are non-homotopic for any starting point including *κ*_0_ (gray point) (which is considered for the example), since they cannot be deformed into one another without crossing EP_3_. Their corresponding matrix product is *M*_1_*M*_2_*M*_1_*M*_2_ and *I*, respectively. This is confirmed by their eigenvalue trajectories as shown in **b** and **c**. **d** The two similar loops ${\bigcirc{{\hskip -6.7pt}{\scriptstyle{3}}}}{\hskip 2pt}$ and ${\bigcirc{{\hskip -6.7pt}{\scriptstyle{4}}}}{\hskip 2pt}$ are non-homotopic for the starting point *κ*_0_ but homotopic for *κ'*_0_. This is also reflected in the exchange relations of the eigenvalues as shown in **e** and **f**. Black dots represent EPs, red lines are the BCs, and the blue loops are the encircling trajectories

We further elucidate on the topological features of equivalent loops in the context of the example given by Eq. (6). In this case, the space *S* would be the space spanned by Re[*κ*] and Im[*κ*] after removing the points EP_1,2,3_ and EP'_1,2,3_. By inspecting the two loops ${\bigcirc{{\hskip -6.7pt}{\scriptstyle{1}}}}{\hskip 2pt}$ and ${\bigcirc{{\hskip -6.7pt}{\scriptstyle{2}}}}{\hskip 2pt}$ in Fig. 5a, it is clear that they are not homotopic for the starting point *κ*_0_. Indeed the net permutation matrix associated with loop ${\bigcirc{{\hskip -6.7pt}{\scriptstyle{1}}}}{\hskip 2pt}$ is *M*_1_*M*_2_*M*_1_*M*_2_, resulting in {*s*_1_, *s*_2_, *s*_3_, *s*_4_} → {*s*_4_, *s*_3_, *s*_2_, *s*_1_}. However, the permutation matrix associated with loop ${\bigcirc{{\hskip -6.7pt}{\scriptstyle{2}}}}{\hskip 2pt}$ is *M*_1_*M*_3_*M*_1_*M*_3_ = *I*. Consequently, their exchange relations are in general different as shown in Fig. 5b, c.

Next, we investigate a scenario that highlights the case of free homotopy. The two loops ${\bigcirc{{\hskip -6.7pt}{\scriptstyle{3}}}}{\hskip 2pt}$ and ${\bigcirc{{\hskip -6.7pt}{\scriptstyle{4}}}}{\hskip 2pt}$ in Fig. 5d are similar (enclose the same EPs), yet they are not homotopic for the starting point *κ*_0_, i.e. they cannot be transformed into one another while keeping the starting point fixed and without crossing EP_2_. Thus, the two loops are not necessarily equivalent. Indeed the net redistribution matrix associated with loop ${\bigcirc{{\hskip -6.7pt}{\scriptstyle{3}}}}{\hskip 2pt}$ is *M*_1_, resulting in {*s*_1_, *s*_2_, *s*_3_, *s*_4_} → {*s*_1_, *s*_3_, *s*_2_, *s*_4_}; while for loop ${\bigcirc{{\hskip -6.7pt}{\scriptstyle{4}}}}{\hskip 2pt}$, the permutation matrix is *M*_2_*M*_1_*M*_2_, which gives {*s*_1_, *s*_2_, *s*_3_, *s*_4_} → {*s*_4_, *s*_2_, *s*_3_, *s*_1_}. On the other hand, if we consider the same loops ${\bigcirc{{\hskip -6.7pt}{\scriptstyle{3}}}}{\hskip 2pt}$ and ${\bigcirc{{\hskip -6.7pt}{\scriptstyle{4}}}}{\hskip 2pt}$ but with a different starting point *κ'*_0_, they are homotopic and the net permutation matrix is *M*_1_ for both loops. Figure 5e, f confirms these results.

So far we have discussed the stroboscopic (or static) exchange between the eigenstates as a result of encircling EPs. Whereas this type of evolution can be in general accessed experimentally (see refs. ^24–26^ for the case of second order EPs), recent theoretical and experimental efforts are painting a different picture for the dynamic evolution, showing that the interplay between gain and loss will inevitably break adiabaticity^31–36^. It will be thus interesting to investigate whether the homotopy between the loops (or its lack for that matter) has any impact on the dynamic evolution. Here we do not attempt to answer this question rigorously but will rather consider illustrative examples. To do so, we focus again on the loops ${\bigcirc{{\hskip -6.7pt}{\scriptstyle{3}}}}{\hskip 2pt}$ and ${\bigcirc{{\hskip -6.7pt}{\scriptstyle{4}}}}{\hskip 2pt}$ shown in Fig. 5d, and we perform a numerical integration to compute the dynamical evolution around these loops starting from either *κ*_0_ or *κ'*_0_. As we discussed before, the loops are similar for both initial conditions but homotopic only for the later one. The computational details are presented in the Supplementary Note 1 but the main results confirm our conclusion. When the two loops are homotopic (i.e. when the initial point on the loop is *κ'*_0_) any initial state *s*_*i*_, with *i* = 1, 2, 3, 4, will end up at state *s*_2_ regardless of the considered loop. For similar but non-homotopic loops (i.e. when the initial point on the the loop is *κ*_0_), the initial states on loop ${\bigcirc{{\hskip -6.7pt}{\scriptstyle{3}}}}{\hskip 2pt}$ always evolve to *s*_3_ while those on loop ${\bigcirc{{\hskip -6.7pt}{\scriptstyle{4}}}}{\hskip 2pt}$ will evolve to *s*_1_. These results suggest that homotopy between the loops plays a much greater role than just describing the static exchange between the states.

**Fig. 6:**
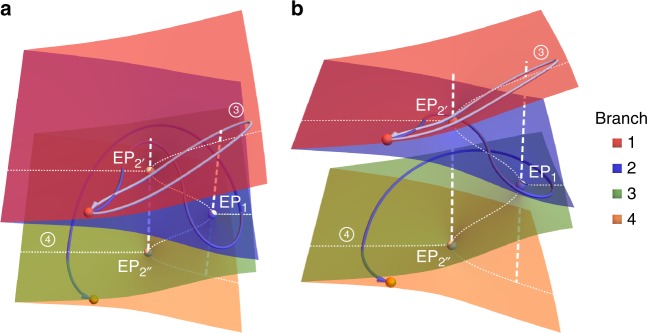
Riemann surface and homotopy between loops. Two different perspectives for the four-sheet Riemann surface (associated with the real parts of the eigenvalues) that corresponds to Fig. 5d are depicted in **a** and **b**. The two loops ${\bigcirc{{\hskip -6.7pt}{\scriptstyle{3}}}}{\hskip 2pt}$ and ${\bigcirc{{\hskip -6.7pt}{\scriptstyle{4}}}}{\hskip 2pt}$ (blue lines) that encircle EP_1_ (white point) in the two-dimensional (2D) parameter space are also shown. As explained in the text, the homotopy test (performed parameter space) for these two loops shows that they are not equivalent, which results in different stroboscopic and dynamic features. On the Riemann surface, this property becomes even more evident by noting that the two loops span different sheets. The red point stands for eigenvalue *λ*_1_ (corresponding to eigenstate *s*_1_) at the initial parameter point. This state will evolve to itself or to the orange point along loops ${\bigcirc{{\hskip -6.7pt}{\scriptstyle{3}}}}{\hskip 2pt}$ and ${\bigcirc{{\hskip -6.7pt}{\scriptstyle{4}}}}{\hskip 2pt}$, respectively. The dashed white lines are vertical lines emanating from the exceptional points (EPs) to illustrate the fact that the projections of the two loops considered here encircle EP_1_ but not EP_2_. The white dotted lines illustrate the eigenvalue bifurcation across the EPs on the Riemann surface

Finally, in order to gain more insight into the topological equivalence (or nonequivalence) between loops that encircle the same EPs and how they affect the dynamic evolution, we plot the Riemann surface that corresponds to Fig. 5d together with the stroboscopic loops ${\bigcirc{{\hskip -6.7pt}{\scriptstyle{3}}}}{\hskip 2pt}$ and ${\bigcirc{{\hskip -6.7pt}{\scriptstyle{4}}}}{\hskip 2pt}$ in Fig. 6. As we have seen before, these two loops encircle the same EP starting from *κ*_0_ yet they are not equivalent. This feature becomes more transparent when we consider the full 3D Riemann surface with its four sheets. Particularly, we see that EP_2_ unfolds into two different points EP_2′_ and EP_2′′_ connecting different sheets. As a result, two trajectories starting from the same point can evolve on different manifolds in 3D space despite the fact that their projection in the 2D parameter space will always encircle the same EP. This in turn explains the difference in the dynamic evolution associated with the two loops. From a practical perspective, these results are very important in the following sense. Recently, the chirality of dynamic encircling of EPs was demonstrated experimentally and studied theoretically. It was shown that the dynamic evolution is very robust, which can be potentially useful for several applications such as non-reciprocal light propagation^43^ and one-way polarization conversion^34^. Our results indicate that when considering more complicated devices, one must take into account the complex EP landscape before making any statement about robustness of the dynamics.

## Discussion

**Fig. 7:**
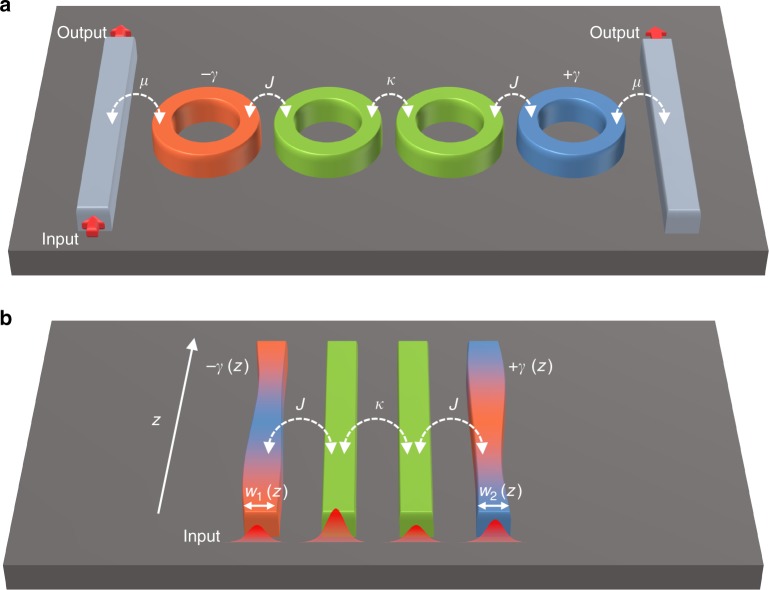
Photonic implementations. Possible photonic platforms for implementing and testing the encircling of multiple exceptional points (EPs) in **a** microring resonators; and **b** waveguides. The gain/loss can be controlled via the pumping (represented schematically by different colors, orange for gain, blue for loss, and  green for neutral.) while the coupling between adjacent elements can be tailored by engineering the edge-to-edge distance. Finally the resonant frequency (or propagation constants) can be tuned by varying the resonator (waveguide) dimensions. For stroboscopic encircling, several samples have to be fabricated, each of which corresponds to a different operating point. The eigenvalues are then plotted and connected smoothly to form the adiabatic loop as having been done before^24–26^. The dynamic encircling on the other hand requires changing the parameter of one sample as a function of time (distance) in resonators (waveguides) platforms. Panel **b** illustrates how this can be achieved in waveguides. Particularly, the propagation constants can be varied along the prorogation distance *z* by changing waveguide dimensions, such as the width for example. The gain/loss can be controlled along *z* by engineering the spatial profile of the optical pump. White arrows indicate the coupling while red thick arrows represent the input/output signal

Here we discuss some possible implementations to observe some of the exotic effects studied in this work. In general several platforms such as photonics, acoustics, optomechanics, microwaves, and electronics can be used. For sake of clarity, here we restrict our discussion to photonic systems. The implementation of the Hamiltonian *H* can be achieved by using four coupled resonators or waveguides as depicted schematically in Fig. 7a, b. From the mathematical point of view, these two systems are equivalent. In the resonator arrangement, the gain and loss coefficients can be varied with time by changing the pumping beam and the system can be probed by studying the scattering coefficients under certain inputs, which can be experimentally implemented by coupling the outer resonators to waveguides as shown in Fig. 7a. On the other hand, the waveguide structure provides more control since, in addition to varying the gain/loss as a function of the propagation distance *z*, here one can also vary the real-valued coupling coefficients (by engineering the distance between the adjacent waveguides) as well as the propagation constants of the waveguides (by tuning the width or height of the guiding channels). Some of these ideas have been recently already explored in refs. ^42,43^. Notably, photonic implementations of the work in ref. ^43^ (dealing with systems that exhibit only one EP) has been recently reported^44^. Further progress will thus enable the experimental investigations of systems having multiple EPs similar to those studied here.

To confirm that these control parameters (gain, loss, propagation constants, and real coupling coefficients) provide enough degrees of freedom to observe the exotic effects, we have investigated the encircling of EPs associated with the Hamiltonian *H* when the coupling remains constant while changing only the gain/loss values of the outermost waveguides and their propagation constants, i.e. the real and imaginary parts of *γ*, respectively (see Supplementary Note 2).

Note that for the stroboscopic encircling, one needs to build different samples, each of which is tuned to a single operating point. The eigenvalues are then measured and plotted to study the exchange relations. On the other hand, one sample with parameters that vary with distance suffices to study the dynamic encircling of EPs as has been shown in refs. ^35,36^ for simple systems having only one EP.

In conclusion, we have introduced a general formalism based on permutation groups and representation theory for describing the stroboscopic encircling of multiple EPs. By using this tool, we uncovered the following counterintuitive results: trajectories that enclose the same EPs starting from the same parameters and traveling in the same direction, do not necessarily result in an identical exchange between the states. Instead, we have shown that this equivalence can be established only between homotopic loops. Additionally, we have also discussed the implication of these results for the dynamic encircling of EPs. Our work may find applications in various fields including the recent interesting work on the relationship between EPs and topological edge states^45,46^. Finally, we would like to comment on potential experimental platforms that can be used to observe the effects discussed here. In principle, any non-Hermitian system where the Hamiltonian parameters can be controlled is a good candidate. This includes optics, microwave, electronics, exciton polaritons, and acoustics^20,21^.

## Electronic supplementary material

Supplementary Information

## Data Availability

The data that support the findings of this study are available from the corresponding authors upon reasonable request.
