# Supplementary material for: Winding around non-Hermitian singularities
Source: Nat Commun. 2018 Nov 15;9:4808. doi: 10.1038/s41467-018-07105-0 (PMC6237871; doi:10.1038/s41467-018-07105-0)
Supplement: Supplementary file 1 — Supplementary Information [file 41467_2018_7105_MOESM1_ESM.pdf]

Supplementary Information:
Winding around Non-Hermitian Singularities

Zhong et al.

Supplementary Note 1. Numerical calculation of dynamic evolution

Here we present the details of the numerical calculations for the dynamic evolution. First, we choose the point $\kappa_0 = (0.4, -0.15)$ in Supplementary Fig. 1. Next, choose the loop ④ in Supplementary Fig. 1a as:

$$\mathrm{Re}[\kappa(\tau)] = \begin{cases} c_1 + r_1\cos(\omega\tau), & \tau \in [0, T/4) \\ c_2 + r_2\cos(\omega\tau), & \tau \in [T/4, T/2) \\ c_1 - r_2\cos(\omega\tau), & \tau \in [T/2, 3T/4) \\ c_3 + r_2\cos(\omega\tau), & \tau \in [3T/4, T] \end{cases}, \tag{1a}$$

$$\mathrm{Im}[\kappa(\tau)] = \begin{cases} r_1\sin(\omega\tau), & \tau \in [0, T/4) \\ r_2\sin(\omega\tau), & \tau \in [T/4, T] \end{cases}, \tag{1b}$$

where $c_1 = 0.7$, $c_2 = 0.4$ and $c_3 = 1$. Note that the centers of the semicircles associated with loop ④ in Supplementary Fig. 1a are given by the coordinates $(c_{1,2,3}, 0)$. The associated radii are $r_1 = 0.45$ and $r_2 = 0.15$. The quantity $T = 4\pi/\omega$ is the time needed to complete one cycle. The exact position of point κ_0' can be now chosen to be the intersection between the line passing through κ_0 and EP_1 and the top large semi-circle, and $\kappa_0' \approx (1.148, 0.03711)$.

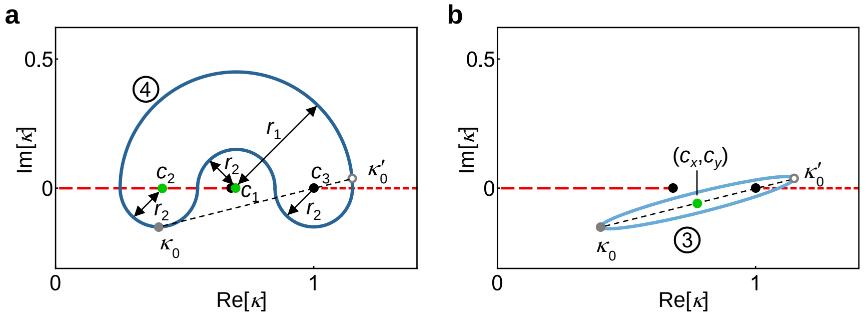

Supplementary Fig. 1. Trajectories of dynamical evolutions. The details of loops ③ and ④ (blue lines) used in the numerical simulation of dynamic evolution of eigenstates in the main text are illustrated in **a** and **b**. Loop ③ is a titled ellipse with the line connecting κ_0 and κ_0' (solid and hollow gray points) as the major axis. The center of ellipse is at (c_x, c_y) (the green point). Loop ④ is a combination of one large semi-circle and three identical small semi-circles. The centers of semi-circles are labeled as $c_{1,2,3}$ with green points and c_3 locates at one exceptional point (EP). The radii are lebeled as $r_{1,2}$.

Finally, loop ③ in Supplementary Fig. 1b was chosen to be a titled ellipse with the line connecting κ_0 and κ_0' as the major axis. This ellipse has semi-major axis $a \approx 0.3858$, focal distance $c = a - 0.002$ and a rotating angle $\theta = \arctan \frac{1}{4}$. Therefore the parametric function of loop ③ is:

$$\mathrm{Re}[\kappa(\tau)] = c_x + a\cos(\omega\tau)\cos\theta - b\sin(\omega\tau)\sin\theta, \tag{2a}$$

$$\mathrm{Im}[\kappa(\tau)] = c_y + a\cos(\omega\tau)\sin\theta + b\sin(\omega\tau)\cos\theta, \tag{2b}$$

where $b = \sqrt{a^2 - c^2}$ is the semi-minor axis of the ellipse and $(c_x, c_y) = (c_2 + a\cos\theta, -r_2 + a\sin\theta)$ is the center of the ellipse.

In all simulations, we chose the encircling speed $\omega = 10^{-4}$. For each loop, the encircling in the counterclockwise/clockwise is performed by the parametrization $\tau = t_0 \pm t$, with $t \in [0, T]$ for loop ④, and $t \in [0, T/2]$ for loop ③ . Here $\tau = t_0$ corresponds to the relevant starting point: for points κ_0 and κ_0', $t_0 = \frac{3T}{8}, \frac{65T}{10^4}$ for loop ④; and $t_0 = \frac{T}{4}, 0$ for loop ③.

Supplementary Note 2. Varying gain/loss instead of coupling

To confirm that these control parameters (gain, loss, propagation constants and real coupling coefficients) provide enough degrees of freedom to observe the exotic effects discussed in the main text, we briefly investigate the encircling of EPs associated with the Hamiltonian H again but this time we fix the couplings and change only the gain/loss parameters of the outermost waveguides and their propagation constants, i.e. the real and imaginary parts of the parameter γ, respectively.

Supplementary Fig. 2a shows two different loops in the parameter space spanned by $\mathrm{Re}[\gamma]$ and $\mathrm{Im}[\gamma]$. Both loops encircle only EP_1, yet they are topologically inequivalent since: Loop ② cannot be deformed into loop ① without crossing EP_2; and the permutation matrices M_1 and M_2 (which are, incidentally, similar to those defined in Eq. (5) associated with $\mathrm{EP}_{1,2}$ in the main text) do not commute. Thus, indeed a system of four coupled waveguides with variable gain/loss and propagation constants can be used to study the topological equivalence between encircling loops in the parameter space.

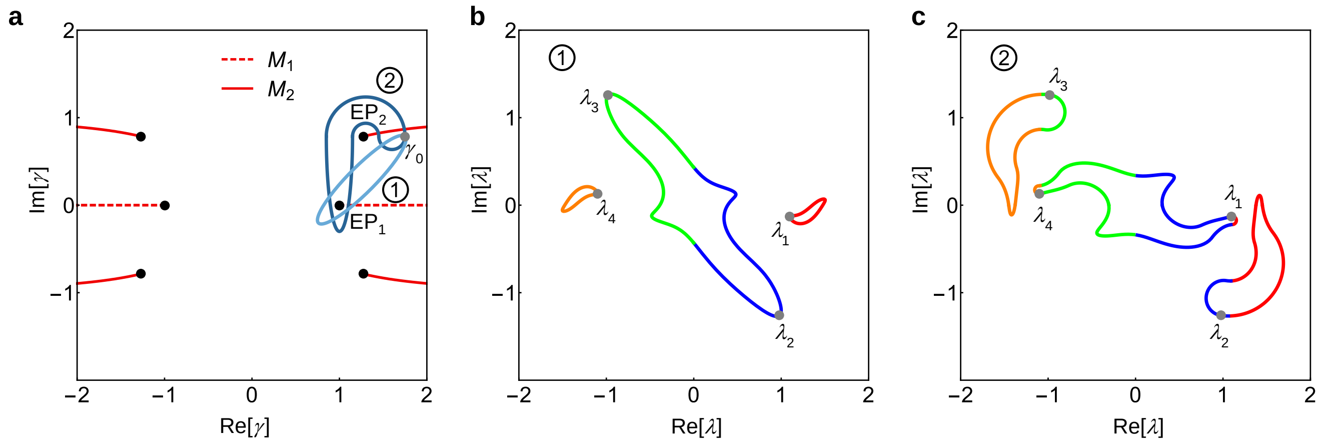

Supplementary Fig. 2. Case study using complex propagation constants. In the example considered in the previous section, we have studied H as we vary the complex coupling coefficients. While this is not impossible, it is rather difficult to achieve experimentally. An easier approach that lends itself to an easier experimental implementation is to change the complex propagation constant which corresponds to changing the real propagation constants and the gain/loss factors. Here we confirm that the main features of this work can be still observed under these conditions. **a** The exceptional points landscape of H in a two-dimensional parameter space spanned by Re[γ] and Im[γ]. One can identify two topologically inequivalent loops (blue lines) that encircle the exceptional point EP$_1$. **b** and **c** show the eigenvalue exchange relations associated with these two loops, confirming their nonequivalence. Black dots represent exceptional points, red lines are the branch cuts. The colors along the eigenvalue trajectory indicate the branch at which the relevant eigenvalue is located.
